# Two Cases of Right‐Sided Pleural Effusion as the Initial Manifestation of Severe Ovarian Hyperstimulation Syndrome

**DOI:** 10.1155/crog/6641371

**Published:** 2026-09-28

**Authors:** Lonneke H. C. Gremmen, Hein Bogers, Catharina C. M. Beerendonk

**Affiliations:** ^1^ Department of Reproductive Medicine, Radboud University Medical Centre, Nijmegen, the Netherlands, radboudumc.nl

**Keywords:** case report, in vitro fertilisation, ovarian hyperstimulation syndrome, thoracentesis, unilateral pleural effusion

## Abstract

**Introduction:**

Severe ovarian hyperstimulation syndrome (OHSS) occurs less frequently due to increased knowledge of potential risk factors and implementation of preventive strategies. Right‐sided pleural effusion as a presentation of severe OHSS is rare, and the symptoms can be nonspecific, which may delay diagnosis. However, early recognition is essential, as the condition can deteriorate rapidly and patients often need hospitalisation and supportive treatment.

**Case Presentation:**

We describe two patients with right‐sided pleural effusion as the initial manifestation of severe OHSS following controlled ovarian stimulation for in vitro fertilisation. Both patients reported sharp chest pain and progressive dyspnoea when the pleural effusion was diagnosed. They developed symptoms 5 and 13 days after oocyte pick‐up (OPU) and were admitted to hospital 4 and 3 days after the onset of symptoms, respectively. The first patient required two paracenteses and one thoracentesis, and the second one thoracentesis. Supportive treatment during hospitalisation consisted of intravenous fluids, supplemental oxygen, electrolyte correction and thromboprophylaxis. The durations of hospitalisation were 12 and 2 days, respectively, and both patients recovered within 3 weeks after discharge.

**Conclusion:**

Right‐sided pleural effusion may be the initial manifestation of severe OHSS. Although the exact pathogenesis remains unclear, several hypotheses have been proposed. We review the available evidence regarding its pathogenesis and therapeutic options, and highlight the importance of early recognition.

## 1. Introduction

Ovarian hyperstimulation syndrome (OHSS) is a well‐known iatrogenic complication of assisted reproduction techniques caused by an excessive ovarian response to stimulation after the administration of human chorionic gonadotropin (hCG). The early form occurs within 9 days after hCG administration and often resolves spontaneously as serum hCG levels decrease. OHSS presenting more than 9 days after hCG administration, usually around 12–17 days, is classified as late OHSS. The late form often has a more severe course as a result of endogenous hCG production by the syncytiotrophoblast.

The exact pathogenesis of OHSS is not fully known, but increased vascular permeability and neovascularisation seem to play a significant role. It is suggested that vasoactive peptides–such as vascular endothelial growth factor (VEGF) and cytokines (e.g., interleukins 1, 2, 6)–enter the systemic circulation through the serosal vessels or the corpus luteum, and are released into the peritoneal cavity by the ovarian prorenin–renin–angiotensin system [1–4].

Risk factors for the development of severe OHSS include young age (< 35 years), polycystic ovary syndrome, previous history of OHSS, high initial antral follicle count (AFC > 24), a large number of follicles prior to ovulation induction (> 17 follicles measuring > 10 mm), retrieval of more than 15 oocytes, oestradiol levels of more than 3500 pmol/L at the time of ovulation trigger and anti‐Müllerian hormone (AMH) levels above 3.4 ng/mL [5, 6].

Various strategies are used to prevent severe OHSS. Primary preventive measures include the use of GnRH antagonist protocols in high‐risk patients and individualised gonadotrophin dosing based on ovarian reserve markers. Secondary strategies include cycle cancellation in long GnRH agonist protocols, GnRH agonist triggering instead of hCG in GnRH antagonist protocols and elective cryopreservation of all embryos. Furthermore, in selected patients, cabergoline may also be considered to reduce the risk of OHSS [7–9].

Mild forms of OHSS occur in 20%–33% of in vitro fertilisation (IVF) cycles, whereas severe OHSS occurs in only 0.1%–2% [10]. Severe OHSS is often characterised by substantial amounts of ascites. In a minority of cases, it is accompanied by pleural effusion, pericardial fluid, renal dysfunction or thromboembolic events. In this article, we present two cases of unilateral pleural effusion as the initial presentation of severe OHSS [9, 11].

## 2. Case Reports

### 2.1. Case 1

A 27‐year‐old woman (gravida 0), with no relevant medical history, presented with primary infertility for 3 years. She had a regular menstrual cycle of 30 days, with an antral follicle count of nine and seven in the right and left ovary, respectively. The semen analysis of her partner showed azoospermia, and they agreed to undergo intracytoplasmic sperm injection (ICSI) using sperm obtained by testicular sperm extraction (TESE).

An agonist gonadotrophin‐releasing hormone (GnRH) protocol was chosen. For planning purposes, an oral contraceptive pill was started on the first day of menstruation for a total duration of 15 days. On Day 12 of the oral contraceptive pill use, a GnRH agonist (triptorelin, Decapeptyl) was added. Following withdrawal bleeding, 13 days after the start of triptorelin, 150 IU/day recombinant FSH (follitropin, Gonal‐F) was initiated. Follicular growth was closely monitored by vaginal ultrasound. With six follicles exceeding 16 mm, and the largest measuring 24.6 mm, ovulation induction was triggered by using 10,000 IU urinary hCG (Gonasi). Approximately 36 h later, an oocyte pick‐up (OPU) was performed, yielding seven oocytes. Two follicles could not be punctured due to pain during the procedure.

Subcutaneous administration of progestin (Prolutex) was initiated to support the luteal phase. Three days after the pick‐up, a single embryo transfer was conducted, and four blastocysts were cryopreserved.

Five days after OPU, the patient complained of dyspnoea and sharp chest pain. Her vital signs were within normal range, and electrocardiography (ECG) showed no abnormalities. Laboratory results showed a slightly increased haemoglobin (8.5 mmol/L) and haematocrit (0.40 L/L), with normal serum albumin (30 g/L) and creatinine (53 *μ*mol/L). Transvaginal ultrasound showed enlarged ovaries measuring 69 × 49 mm (right) and 88 × 61 mm (left), with a small amount of free fluid in the rectouterine pouch. The CT angiography showed right‐sided pleural effusion and subdiaphragmatic fluid. The patient was discharged with instructions to maintain a daily fluid intake of at least 2.5 L and contact the department if her condition deteriorated. A follow‐up appointment was scheduled.

The patient presented at the fertility department 4 days later with nausea and worsening dyspnoea. She had an oxygen saturation level of 92%–94% on room air, a normal blood pressure and a heart rate of 100–130 beats per minute. Reduced breath sounds were heard over both lower lung fields. Laboratory results revealed a haemoglobin of 9.7 mmol/L and a haematocrit of 0.46 L/L, suggesting haemoconcentration. Chest X‐ray demonstrated a large right‐sided pleural effusion, which was significantly increased compared to the CT angiogram (Figure 1a). She was admitted and received 2 L/min supplemental oxygen via a nasal cannula and a daily intravenous administration of 2.5 L of 0.9% sodium chloride. To prevent thromboembolism, a low‐molecular‐weight heparin (LMWH) was started. Thoracentesis was performed, removing 1750 mL of fluid from the right pleural space. Pleural fluid analysis demonstrated a pH of 7.6, a high protein concentration (total protein 45.1 g/L; albumin 27 g/L), and an LDH level of 94 U/L (serum LDH 184 U/L). Cytological examination showed a predominance of immune cells and no evidence of malignancy. Bacterial cultures were negative.

**Figure 1:**
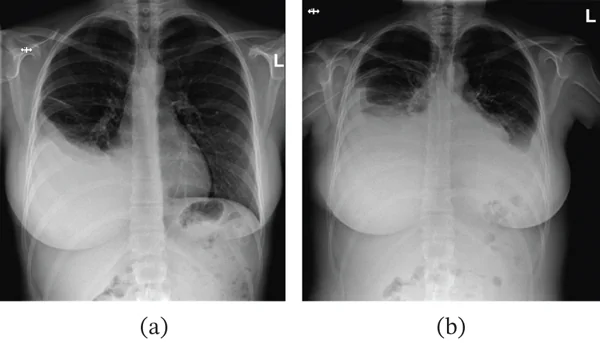
(a) Chest X‐ray obtained 9 days after oocyte pick‐up (OPU), showing a large right‐sided pleural effusion with opacification of the right lower lung field. (b) Chest X‐ray obtained on Day 5 of hospital admission, 14 days after oocyte pick‐up (OPU), showing progression from a unilateral right‐sided pleural effusion to extensive bilateral pleural effusions with opacification of both lower lung fields.

On the third day of admission, the patient complained of abdominal distension. She had gained 5 kg since admission, and her abdominal girth had increased by 6 cm. She had a normal blood pressure, a heart rate of around 105 beats per minute, and oliguria of 20–25 mL per hour. The laboratory results showed severe haemoconcentration, with a haemoglobin of 10.0 mmol/L and haematocrit of 0.47 L/L, as well as hypoalbuminaemia (19 g/L). Transvaginal paracentesis was performed, draining 1330 mL of fluid from the abdominal cavity.

The next day, blood tests revealed hyponatraemia (sodium 132 mmol/L), hypokalaemia (potassium 3.2 mmol/L) and a further decrease in serum albumin (15 g/L). Because of severe nausea, sufficient oral intake could not be maintained. A peripherally inserted central catheter (PICC) was placed to start parenteral nutrition and potassium supplementation (40 mmol/24 h).

Two days after the paracentesis, the patient reported worsening abdominal distension and shortness of breath. She had a heart rate of 120 beats per minute, her oxygen saturation level was 89%–92% despite 3 L/min of oxygen through a nasal cannula, and her weight had increased by an additional 4 kg. The hyponatraemia worsened (sodium 126 mmol/L), and a chest X‐ray showed severe bilateral pleural effusions (Figure 1b). She was transferred to the medium care unit, where an abdominal paracentesis was performed, removing 3300 mL of fluid. The hyponatraemia and hypoalbuminaemia were treated with 100 mL of 3% (0.03 g/mL) sodium chloride over 15 min and 200 mL of 20% (0.2 g/mL) albumin as a rapid intravenous bolus, respectively.

The patient returned to the gynaecology ward 1 day later. She experienced less dyspnoea, and her oxygen saturation level improved to 97% on room air. Over the next few days, oral intake improved, and parenteral nutrition was gradually reduced. On Day 11 of admission, the haemoglobin (8.0 mmol/L) and haematocrit (0.38 L/L) were within normal range, and serum sodium (133 mmol/L) and albumin (27 g/L) levels were only slightly reduced. The PICC was removed, and the patient was discharged the following day. Prophylactic LMWH was prescribed for a total of 3 months.

Over the next 3 weeks, the patient showed clinical improvement. At a gestational age of 8 weeks and 3 days, ultrasound showed a viable intra‐uterine pregnancy with a delayed crown‐rump length (CRL) corresponding to 6 weeks and 3 days. Unfortunately, at 10 weeks and 5 days’ gestation, no foetal heartbeat was detected, and the CRL corresponded to 7 weeks and 3 days. The options of expectant management or medical induction were discussed. After a few days, the patient chose medical induction with mifepristone and misoprostol, which proceeded without complications. Two weeks later, a complete miscarriage was confirmed by ultrasound.

The patient’s clinical course is summarised in Table 1.

**Table 1:** Timeline of the clinical course of both patients.

Event	Case 1	Case 2
Oocyte retrieval	Day 0	Day 0
Onset of OHSS symptoms	Day 5	Day 13
Diagnosis of pleural effusion	Day 5	Day 16
Hospital admission	Day 9	Day 16
Thoracentesis	Day 9	Day 16
Paracentesis	Days 12 and 14	None
Hospital discharge	Day 21	Day 18
Clinical and biochemical recovery	3 weeks after discharge	2 weeks after discharge

### 2.2. Case 2

A 33‐year‐old woman (gravida 0) presented with primary infertility for 2 years, due to endometriosis and a mild male factor. Her medical history included laparoscopic surgery for endometriosis, including cystectomy of a right ovarian endometrioma, drainage of bilateral endometriomas and excision of peritoneal endometriosis. She had oligomenorrhea, and her antral follicle count was 7 in the right ovary and 20 in the left ovary. She had a high AMH level of 16.17 ng/mL. Semen analysis of her partner showed a total motile count (TMC) of nine million with 26% Grade A motility. ICSI using a long GnRH agonist protocol was chosen.

No oral contraceptive pill was started, since the patient received a GnRH agonist (leuprorelin, Lucrin) injection 3 months prior as part of her endometriosis treatment. A GnRH agonist (triptorelin, Decapeptyl) was started, followed by a recombinant FSH (follitropin, Gonal‐F) at a dose of 100 IU/day 2 days later.

When eight follicles exceeded 16 mm, with the largest measuring 20 mm, the ovulation was triggered by using 10,000 IU urinary hCG (choriogonadotropin, Gonasi). The patient received prophylactic amoxicillin/clavulanate (1000 mg/250 mg) prior to OPU because of known ovarian endometriosis. Approximately 36 h later, an OPU was performed, yielding seven oocytes from the left ovary. Multiple small follicles and several larger follicles in the left ovary could not be reached. In the right ovary, a single large follicle was seen, which could not be punctured due to indistinguishable intervening tissue. After OPU, subcutaneous administration of progestin (Prolutex) was started.

Three days later, the patient was seen for embryo transfer. She had no complaints, and physical examination showed no abnormalities. Transvaginal ultrasound showed enlarged ovaries with some free fluid (38 × 41 mm) in the recto‐uterine pouch. Laboratory results were all within normal limits. A single embryo transfer was conducted, and two blastocysts were cryopreserved.

Thirteen days after OPU, the patient complained of dyspnoea, oedema in the lower extremities and an increase in abdominal circumference. She gained 2 kg in weight. Her vital signs were within normal limits, and normal breath sounds were heard. Transvaginal ultrasound showed enlarged ovaries with a maximum diameter of 120 mm, and free fluid in the rectouterine pouch measuring up to 50 mm. Laboratory results showed only a slightly increased haemoglobin (8.7 mmol/L), with a normal haematocrit (0.40 L/L) and creatinine (46 *μ*mol/L). Based on the presence of a small amount of ascites and a slightly increased haemoglobin, the patient was initially diagnosed with mild OHSS. The patient was discharged with instructions to maintain a daily fluid intake of at least 2.5 L and to contact the department if her symptoms worsened.

Three days later, the patient presented with dyspnoea, mainly when lying on the left side, and sharp chest pain when breathing. She had gained 3 kg. She had a decreased oxygen saturation level of 93%–96% on room air, normal blood pressure and a heart rate of 95–110 beats per minute. Reduced breath sounds were heard over the right lower lung field. Transvaginal ultrasound showed an increase in the amount of free fluid in the rectouterine pouch and the upper abdomen. Her haemoglobin had risen to 9.4 mmol/L, with a haematocrit of 0.43 L/L. Both creatinine (65 *μ*mol/L) and serum albumin (33 g/L) were within normal limits. ECG showed no abnormalities. The CT localiser radiograph showed opacification of the right hemithorax (Figure 2), and transthoracic ultrasonography confirmed a large amount of fluid in the right pleural space. A thoracentesis was performed, removing 2000 mL of fluid from the right pleural space. Pleural fluid analysis showed a pH of 7.7, a high protein concentration (total protein 44.2 g/L; albumin 29 g/L), and an LDH level of 108 U/L (serum LDH 171 U/L). Cytological examination demonstrated reactive mesothelial cells, lymphocytes and histiocytes, without evidence of malignancy. Bacterial cultures remained negative.

**Figure 2:**
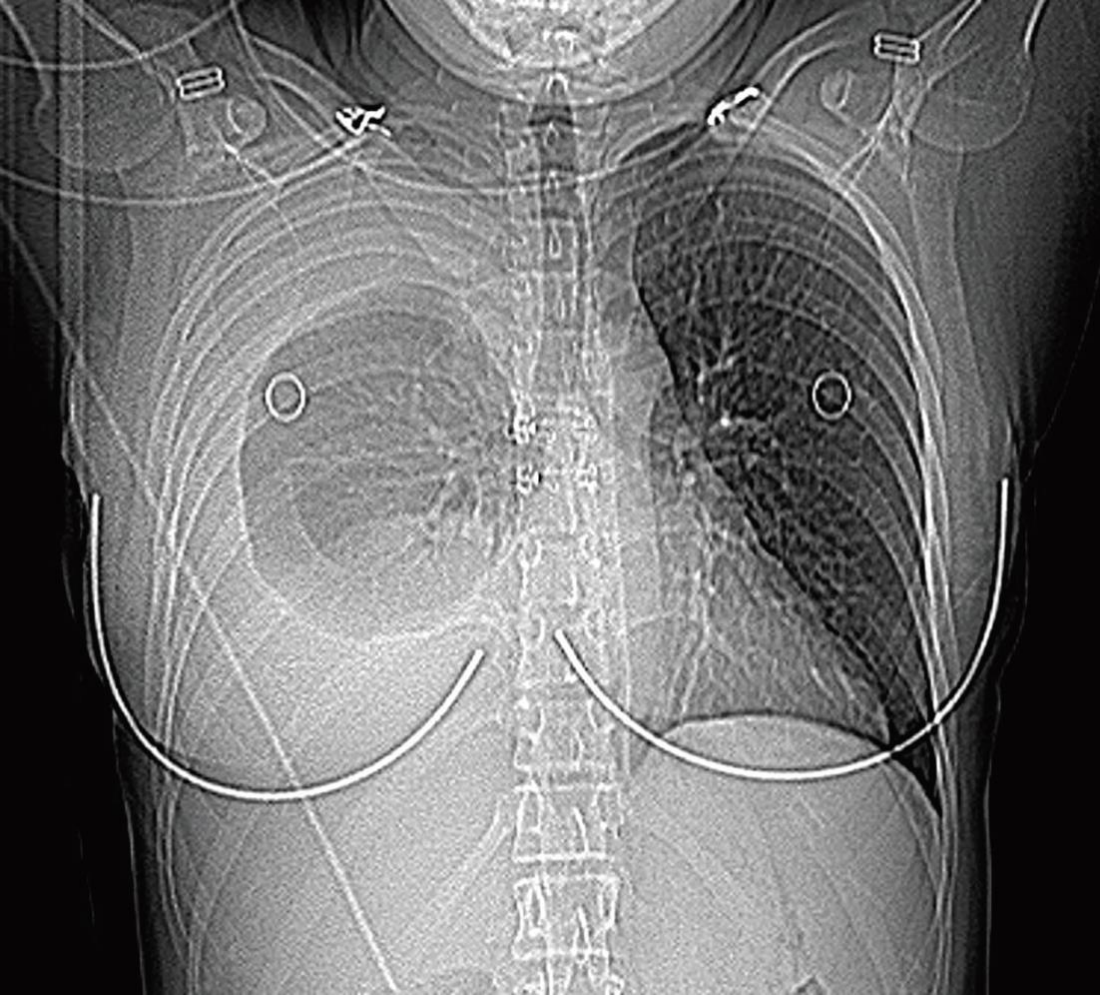
CT localiser radiograph obtained 16 days after oocyte pick‐up (OPU), showing a large right‐sided pleural effusion with associated right lung volume loss.

The patient was admitted and received 2 L of intravenous 0.9% sodium chloride daily. LMWH was started to prevent thromboembolism.

Over the next 2 days, the dyspnoea decreased, and intravenous fluid supplementation was gradually reduced. Her vital signs remained within normal limits, except for a slightly decreased oxygen saturation level of 96% on room air. Decreased breath sounds remained in the lower half of the right lung field. On the day of discharge, 2 days after admission, her haemoglobin (8.4 mmol/L) and haematocrit (0.40 L/L) were within normal ranges, accompanied by a slightly decreased serum albumin level (27 g/L). Prophylactic LMWH was prescribed for a total period of 3 months.

Over the next 2 weeks, the patient was seen for follow‐up at the fertility department. Her dyspnoea gradually decreased, and decreased breath sounds were only heard over the right lower lung field. Laboratory results showed a gradual increase in serum albumin levels, and a transient mild elevation of the liver enzymes. At a gestational age of 7 weeks, a viable intra‐uterine pregnancy with a CRL corresponding to the gestational age was seen.

The patient’s clinical course is summarised in Table 1.

## 3. Discussion

We describe two cases of right‐sided pleural effusion as a rare initial manifestation of severe OHSS. In the first case, two paracenteses and a thoracentesis were performed, draining a total of 6380 mL of fluid. In the second case, a thoracentesis was performed, draining 2000 mL of pleural fluid. Severe and potentially life‐threatening OHSS is uncommon and occurs in approximately 0.1%–2% of IVF cycles [10]. This may be an underestimation of the actual incidence, as there is no consistent definition of severe OHSS. OHSS can be divided into an early and a late form. Early OHSS often resolves relatively fast as serum hCG levels decrease, whereas late OHSS often has a more severe and prolonged course due to sustained endogenous hCG production by the syncytiotrophoblast. Notably, our first case developed early‐onset OHSS, with the first symptoms starting 5 days after OPU. She had a severe and prolonged course despite having few risk factors and no significant symptoms of OHSS at the time of embryo transfer. This may have been the result of increasing endogenous hCG production associated with the pregnancy. In contrast, the second case developed late‐onset OHSS, with the first symptoms starting 13 days after OPU, and showed faster clinical improvement compared with the first case. Cases with severe OHSS are often characterised by a large amount of ascites and, less frequently, by pleural effusion, pericardial fluid, renal dysfunction and thromboembolic events. Current guidelines emphasise individualised preventive strategies in women at high risk of OHSS [7–9]. In our first case, the only known risk factor was age below 35 years. Based on her initial low‐risk profile, a long GnRH agonist protocol with standard doses of recombinant FSH and hCG was chosen. In the second case, the risk factors included age below 35 years, an initial AFC above 24, and an AMH level of 16.17 ng/mL. Because the patient had already received a depot GnRH agonist for endometriosis, a GnRH antagonist protocol was not feasible. Therefore, based on her higher‐risk profile, the dose of recombinant FSH was reduced. In multiple countries, the ‘freeze‐all’ strategy is used in most patients undergoing IVF. However, in our country this approach is not used as standard practice. Since neither patient showed significant symptoms of OHSS before embryo transfer, both patients underwent fresh single embryo transfer in accordance with the Dutch national guideline [7, 8].

Pleural effusion is a rare manifestation of severe OHSS. Unilateral pleural effusions are usually right‐sided [11, 12]. The exact mechanism by which unilateral pleural effusion in OHSS develops is not fully understood, but it is likely multifactorial. One proposed mechanism is the shift of intra‐abdominal fluid into the negatively pressured pleural cavity through defects in the diaphragm. Macroscopic defects in the tendinous part of the diaphragm have been described during thoracic surgery and postmortem examinations. These defects are more common in women and are more often seen on the right side, which may explain why approximately 80% of unilateral pleural effusions in OHSS are right‐sided [13–15]. A similar right‐sided predominance of diaphragmatic lesions has been described in endometriosis and ovarian cancer. The tendency of intraperitoneal fluid to flow along the right paracolic gutter and right hemidiaphragm may play a role [16, 17]. Another possible mechanism is leakage of fluid through the diaphragmatic lymphatic vessels. This mechanism has also been described in other conditions associated with both ascites and pleural effusion, such as liver cirrhosis and Meigs’ syndrome [14, 15]. Lastly, increased permeability of vessels in the pleural serosa may play a role. However, lymphatic leakage and increased pleural vascular permeability are likely to affect both pleural cavities and therefore may not fully explain why unilateral pleural effusions in OHSS are predominantly right‐sided [18–22].

The composition of pleural fluid may provide additional information about the underlying pathophysiology. A systematic review by Irani et al. reported that 66% of pleural effusions in patients with severe OHSS were classified as transudative, and the remaining 33% as exudative [11]. Pleural effusions are classified as exudative when one or more of the three Light’s criteria are met. In our cases, the pleural fluids of both patients contained high protein concentrations (4.5 and 4.4 g/dL), suggesting an exudative component. However, the Light’s criteria fluid‐to‐serum protein ratio could not be determined because the total serum protein concentrations were not available. The second criterion, based on the pleural fluid LDH concentrations (94 and 108 U/L), was not met in either patient, suggesting a transudative component. The pleural fluid‐to‐serum LDH ratios (0.51 and 0.63) suggested transudative and exudative characteristics, respectively. Evaluation of pleural fluid using Light’s criteria may help classify pleural effusions as exudative or transudative. However, both exudative and transudative characteristics were observed in our cases.

A systematic review by Irani et al. showed that 90% of patients with severe OHSS and pleural effusion required thoracentesis [11]. However, given the risk of complications, such as pneumothorax, and the potential need for repeated procedures, thoracentesis should be reserved for patients with severe symptomatic pleural effusion. Paracentesis may also improve respiratory symptoms by reducing intra‐abdominal pressure. Current evidence is insufficient to recommend an optimal drainage volume. The current Dutch OHSS guideline recommends draining enough ascites to relieve symptoms, as long as this can be performed safely. Given the risk of significant fluid shifts and the relatively high recurrence rate, paracentesis should be considered in patients with severe symptomatic ascites or haemodynamic compromise [7].

Human albumin is derived from human plasma and has a half‐life of approximately 20 days. It increases intravascular volume by raising the plasma oncotic pressure. The use of albumin supplementation in reproductive medicine has mainly been studied for prophylactic purposes. Initial research indicated that intravenous albumin at the time of oocyte retrieval might decrease the rate of severe OHSS in high‐risk women [23]. However, recent studies were unable to replicate these findings, and multiple guidelines recommend against the use of prophylactic albumin supplementation [7, 8, 24]. Some guidelines mention the therapeutic use of human albumin to treat hypoalbuminaemia and hypovolaemia in patients with OHSS [7, 9]. However, the effectiveness of albumin supplementation in OHSS has not been sufficiently demonstrated. In addition, evidence regarding the safety and cost‐effectiveness of albumin supplementation in this population is lacking [25, 26]. Our first patient received intravenous albumin during abdominal paracentesis, after which her clinical condition improved. However, based on a single case, no conclusions can be drawn regarding the contribution of albumin supplementation to the observed improvement. Studies are needed to determine whether patients with OHSS and severe hypoalbuminaemia benefit from albumin supplementation, to identify the patients who are most likely to benefit, and to establish optimal timing, dose and duration.

These cases provide insight into a very rare manifestation of severe OHSS. However, the ability to draw conclusions about the underlying pathophysiology and the effectiveness of treatment is limited, as we describe only two patients.

## 4. Conclusion

We describe two cases of right‐sided pleural effusion as a rare initial manifestation of severe OHSS. The underlying mechanism remains unclear, but may involve passage of ascitic fluid into the pleural cavity through diaphragmatic defects, increased permeability of the pleural vessels or leakage through the diaphragmatic lymphatic vessels. Evaluation of pleural fluid using Light’s criteria may help classify pleural effusions as exudative or transudative. However, the pleural fluid in our cases showed both exudative and transudative characteristics, reflecting the complex pathophysiology of pleural effusion in OHSS. Most patients with severe OHSS and pleural effusion require thoracentesis, and paracentesis can also be considered to reduce respiratory symptoms. The role of albumin supplementation in the treatment of severe OHSS remains unclear, and studies are needed to define its place in clinical practice.

## Author Contributions

**Lonneke H. C. Gremmen:** investigation; data curation; writing – original draft; visualization.

**Hein Bogers:** conceptualization; supervision; writing – review and editing.

**Catharina C. M. Beerendonk:** supervision; resources; writing – review and editing.

## Funding

No funding was received for this manuscript.

## Disclosure

All authors have read and approved the final version of the manuscript. Lonneke H. C. Gremmen had full access to all of the data in this study and takes complete responsibility for the integrity of the data and the accuracy of the data analysis.

## Ethics Statement

Ethical approval was not required for this report because it describes two anonymised clinical cases. Written informed consent for publication of the case report and the accompanying radiological images was obtained from both patients.

## Conflicts of Interest

The authors declare no conflicts of interest.

## Data Availability

The authors confirm that the data supporting the findings of this study are available within the article.
